# Depression and Violence in Adolescence and Young Adults: Findings From Three Longitudinal Cohorts

**DOI:** 10.1016/j.jaac.2017.05.016

**Published:** 2017-08

**Authors:** Rongqin Yu, Mikko Aaltonen, Susan Branje, Tiina Ristikari, Wim Meeus, Katariina Salmela-Aro, Guy M. Goodwin, Seena Fazel

**Affiliations:** a University of Oxford, Oxford, UK; b University of Helsinki, Helsinki, Finland; c Utrecht University, Utrecht, The Netherlands; d National Institute for Health and Welfare (THL), Oulu, Finland; e Utrecht University and Tilburg University, Tilburg, The Netherlands; f University of Jyväskylä, Jyväskylän, Finland

**Keywords:** depression, violence, adolescents and young adults, longitudinal, birth cohort

## Abstract

**Objective:**

Despite recent research demonstrating associations between violence and depression in adults, links in adolescents are uncertain. This study aims to assess the longitudinal associations between young people’s depression and later violent outcomes.

**Method:**

We used data from three cohorts with different measurements of depression exposures and subsequent violent outcomes. In a Dutch community cohort Research on Adolescent Development And Relationships (RADAR; N = 623) and a population-based British birth cohort Avon Longitudinal Study of Parents and Children (ALSPAC; N = 4,030), we examined the longitudinal links between adolescent depressive symptoms and violent behaviors from age 13 to 17 years. In a total Finnish birth cohort (FBC 1987; N = 57,526), we estimated risk of violent convictions in individuals clinically diagnosed with depression from age 15 to 27 years.

**Results:**

During a mean follow-up period of 4 years, the adjusted odds ratio (aOR) of violent behaviors per unit of increase in depressive symptoms was 1.7 (95% CI = 1.2–2.5) in the Dutch RADAR community sample and 1.8 (95% CI = 1.4–2.3) in the British ALSPAC birth cohort. In the FBC 1987 cohort, the aOR of violent convictions was 2.1 (95% CI = 1.7–2.7) among individuals with a depression diagnosis compared with general population controls without depression. All risk estimates were adjusted for family socioeconomic status and previous violence.

**Conclusion:**

Consistent findings across three longitudinal studies suggest that clinical guidelines should consider recommending risk assessment for violence in young people with depression. The benefits of targeting risk management in subgroups by gender need further investigation.

Recent empirical work has shown a heightened risk of violence in individuals with depression. This depression–violence link in adults has been reported in cross-sectional community surveys and register-based investigations,^1^, ^2^ as well as longitudinal cohort studies.^3^, ^4^, ^5^ In particular, a recent longitudinal investigation of the whole Swedish population reported that the risk of violent crime in individuals with depression was around three times higher than in the normal population and increased twofold compared with that in their siblings without depression.^4^ However, research into the depression–violence link currently lacks a developmental perspective. The link might even be stronger in adolescents than in adults because adolescent depression, compared with its adult form, is more likely to present with temper tantrums and irritability,^6^ which may mediate any association with violence. Prior research has evaluated the depression–violence link mainly in clinical samples in which depression is evaluated dichotomously, and further clarification is required to determine whether this relationship is maintained at a nonclinical level. This is potentially important because minor depressive symptoms in adolescence predict full-blown depressive disorder and problem behaviors later in life, and are more common than adult depression.^7^ Therefore, research into the depression–violence link in community adolescent samples in which both minor depressive symptoms and violent behaviors are prevalent^8^, ^9^ will address current uncertainties.

In addition, high rates of depression have been reported among adolescents in juvenile detention and correctional facilities (which have been estimated at 11% in boys and 29% in girls).^10^ Despite the fact that that depression may co-occur with violence or related disorders such as conduct disorders,^11^ and that factors such as genetic vulnerabilities^12^ or peer victimizations^13^ have been suggested to mediate these links, clarifying the longitudinal association between depression and subsequent violence among young people is required with careful adjustment for confounding factors. Research in adolescents could inform primary prevention and clinical management in particularly sensitive developmental periods for assessing and managing violence risk in depression. Adolescent depression is often underdiagnosed and untreated until later in life.^9^, ^14^ If a longitudinal association between adolescent depression and violence is identified, prevention efforts could further be shifted to adolescents, especially because there is clear evidence of effectiveness of treatment in this developmental period.^15^

Therefore, we have analysed data from three longitudinal studies to investigate links between depression and violence using both community and clinical samples of adolescents and young adults. Data from three European countries (i.e., the Netherlands, the United Kingdom, and Finland) were used. The epidemiological rates of depression and violence appear to be broadly similar in these three countries, with the prevalence of depressive disorders ranging from 2.5% to 3.6%^16^ and violent convictions around 3% among young people.^17^, ^18^

## Method

### Study Samples

We adopted a multiple-sample approach to examine the depression–violence link. We used three validated longitudinal datasets: a Dutch community cohort (Research on Adolescent Development And Relationships [RADAR]; N = 678); a population-based birth cohort in the UK (Avon Longitudinal Study of Parents and Children [ALSPAC]; N = 13,973) born in 1991 or 1992; and a total birth cohort of Finns born in 1987 (the 1987 Finnish Birth Cohort [FBC 1987]; N = 59,476). These three samples were selected for a number of major reasons. First, all of the cohorts included high-quality longitudinal data on depression and violence in young persons from a small-scale community sample to a total birth cohort. Second, these three cohorts used complementary measures of the examined variables, including self-report and clinical diagnoses of depression, and self/informant-report of violent behaviors and official registration of violent convictions. The variation in the sample characteristics and complementary measures of the studied variables provided an opportunity to test the link between depression and violence more comprehensively than previously done.

RADAR is an ongoing longitudinal study in the Netherlands. Adolescents were recruited from randomly selected schools in the midwestern part of the Netherlands. Data from 623 Dutch adolescents (341 male [55%]) who were annually followed from age 13 to 17 years were used. The mean age of the sample was 13.1 years (SD = 0.5 year) at first measurement. The majority of the participants identified themselves as Dutch (n = 499 [80.1%], with the other 124 participants [19.9%] being ethnic minorities). The RADAR cohort study was approved by the ethical-medical committee of University Medical Centre Utrecht, the Netherlands.

ALSPAC is a population-based birth cohort study in the UK. ALSPAC recruited 15,247 pregnant women residents in the Avon area who expected to deliver a child between 1 April 1991 and 31 December 1992, resulting in 15,458 children. For reasons of confidentiality, triplets and quadruplets were excluded. In the current study, we used data from a subsample of adolescents who participated in ALSPAC clinic sessions and were asked to fill in questionnaires related to depressive symptoms and violent behaviors. The total sample included 4,030 adolescents (1,768 male [43.9%]). Ethical approval for the study was obtained from the ALSPAC Ethics and Law Committee and the local research ethics committee.

The FBC 1987 study is a prospective nationwide population investigation in Finland. It consists of 60,069 children born in Finland in 1987.^19^ Follow-up information was collected for all cohort members who survived the first week after birth (N = 59,476; 30,435 male [51.2%]). Data on the use of health care services, criminality, as well as family and socio-demographic background were obtained from official registers. The register data from the Finnish Legal Register Centre, Finnish Hospital Discharge Register (HDR), Statistics Finland Registers, and Population Register were combined using the participants’ personal identification numbers. The FBC 1987 cohort study was approved by the ethical committee of the National Institute for Health and Welfare (§28/2009).

### Measures

#### Depressive Symptoms and Diagnosis

In the RADAR and ALSPAC cohorts, depressive symptoms were assessed with questionnaires. In RADAR, Reynolds’s Adolescent Depression Scale (2nd RADS-2)^20^ was used, which is a self-report questionnaire with 23 items (e.g., “I feel like nothing I do helps anymore”). At age 13 years, adolescents responded to the questionnaire on a 4-point Likert-type scale, ranging from 1 (almost never) to 4 (usually). Cronbach’s α of this scale was .93. In the ALSPAC cohort, depressive symptoms were measured by the short version of the Mood and Feelings Questionnaire (SMFQ),^21^ which is a 13-item checklist of symptoms experienced in the previous 2 weeks (e.g., In the past 2 weeks, have you felt miserable or unhappy?). Adolescents reported on their depressive symptoms at age 13 years, with a three-point scale including 1 (true), 2 (sometimes true), and 3 (not true). Data were reversely coded so that higher scores indicated higher depressive symptoms. Cronbach’s α of the scale was .84. Mean scores of depressive symptoms were calculated for the logistic regression analyses.

For FBC 1987, information on clinical diagnoses, rather than symptoms (which was not collected), was obtained from the Finnish Hospital Discharge Register (HDR) held by the National Institute for Health and Welfare (THL). HDR has been found to be a valid and reliable tool for epidemiological research.^22^ The register includes all specialized inpatient and outpatient visits in public hospitals since 1998; participants were on average 11 years of age. Participants with at least two outpatient episodes as a main diagnosis between January 1, 1998, and December 31, 2012 (11–25 years) were identified as having a diagnosis of depression, according to the *International Classification of Diseases–10*^*th*^ *Revision* (*ICD*-*10*; codes F32-F33.9).

Among the 59,476 participants in the FBC 1987 cohort, 3,666 (6.2%) had at least one outpatient (specialist) episode with a clinical depression diagnosis. We excluded participants with only one outpatient visit for a depressive episode (n = 772), or with a secondary diagnosis of schizophrenia, schizophrenia spectrum disorder, or bipolar disorder (n = 74), as those cases were most likely misclassified as depression during the first outpatient visits. We also excluded those participants with an inpatient diagnosis of depression (n = 923), as it would lead to selection biases: individuals’ admissions to the hospital might have been secondary to recent violence or violence risk. In addition, we excluded 262 individuals with an inpatient diagnosis of depression but without an outpatient diagnosis of depression from the general control sample. Furthermore, we excluded participants with no exact date of violent crime (n = 78) from either case or control samples. Therefore, there were 2,050 individuals with clinical depression diagnoses and 55,476 general controls without depression.

#### Violent Behaviors and Convictions

In the RADAR and ALSPAC cohorts, violent behaviors in the past 12 months were measured with questionnaires. In RADAR, a scale based on the International Self-Report Delinquency Study^23^ was used. Violent behaviors included stealing from a person with threat or force, assault, hurting someone with weapon, and beating or kicking someone. Adolescents who committed at least one of the listed violent behaviors from the second to the fifth measurements (14–17 years of age) were categorized as being violent. In ALSPAC, both adolescents and their mothers reported on adolescents’ violent behaviors in a confidential self-reported questionnaire, originally developed in the Edinburgh Study of Youth Transitions and Crime.^24^ Adolescents reported when they were 13, 14, and 17 years of age, and their mothers reported when adolescents were 16 years of age. Adolescents were categorized as being violent if they had committed any of the following behaviors from measures at age 14 to 17: stole from person with force or threats, assaulted, hit, kicked, or punched someone, and used weapons in fights. Violence measured at age 13 years was used as a control variable in RADAR and ALSPAC.

In FBC 1987, information on violent convictions was retrieved from the Finnish Legal Registration Centre that covers crimes committed for all individuals from the age of 15 years, the age of criminal responsibility in Finland, to age 27 (i.e., over the period January 1, 2002 to December 31, 2014). We extracted data for violent crimes both before (as covariate) and after (as outcome) diagnosis of depression. For population controls without depression, violent convictions occurring before and after the mean date of diagnosis of depression were used as covariate and outcome, respectively. Convictions for violent crimes in FBC 1987 included homicide, all forms of assault, robbery, rape, and sexual offense toward a child.

### Statistical Analyses

To calculate the odds ratio of later violence in depression, we conducted logistic regression analyses in M*plus*,^25^ with depressive symptoms and diagnosis as the independent variable and with subsequent violent behaviors and convictions as dependent variables. To examine whether the association between depression and violent outcomes differed between boys and girls, subgroup analyses were conducted.

In regard to confounders, we took into account effects of family socioeconomic status and previous violence in all three cohorts. Controlling for previous violence could inform us as to whether adolescent depression predicted changes in violence over time. In the additional sensitivity analyses, we also adjusted for other factors including parental criminality, conduct disorder, and maltreatment where data were available.

## Results

### Descriptive Statistics

Table 1 presents descriptive statistics for depressive symptoms, depression diagnosis, and violent outcomes in the three longitudinal cohorts. The prevalence of violent behaviors from age 14 to 17 years was 29.7% in the RADAR cohort, based on adolescents’ self-reported violent behaviors in the previous 12 months across four annual waves. In the ALSPAC cohort, the prevalence of violent behaviors from age 14 to 17 years was 35.2%, using both self and mother reports of violence in the previous 12 months. In the FBC 1987 cohort, 3.7% were convicted of violent crimes during 2002 and 2014 (i.e., during ages 15–27 years).

**Table 1:** Descriptive Statistics for Depression and Violence in Adolescents and Young Adults in Three Longitudinal Cohorts

	Depression^a^			Violence^b^		
	Age	Mean (SD)	Range	Age	n	%
RADAR (N = 623)	13.0	1.61 (0.5)	1.0–4.0	14.1–17.1	185	29.7
Male	13.1	1.55 (0.5)	1.0–4.0	14.1–17.1	120	35.2
Female	13.1	1.69 (0.5)	1.0–3.7	14.1–17.1	65	23.0
ALSPAC (N = 4,030)	13.0	1.31 (0.3)	1.0–2.9	14.0–17.0	1,410	35.2
Male	13.0	1.27 (0.3)	1.0–2.9	14.0–17.0	761	43.1
Female	13.0	1.33 (0.3)	1.0–2.9	14.0–17.0	659	29.1
FBC 1987 (N = 57,526)	19.7^c^	2,050 (3.6)^d^	0 or 1	19.6^e^	2,127	3.7
Male	19.8^c^	668 (2.2)^d^	0 or 1	19.5^e^	1,784	6.0
Female	19.6^c^	1,382 (5.0)^d^	0 or 1	19.6^e^	343	1.2

Note: ALSPAC = Avon Longitudinal Study of Parents and Children; FBC = Finnish Birth Cohort; RADAR = Research on Adolescent Development And Relationships.

a Self-reported depressive symptoms in RADAR and ALSPAC and clinical diagnosis of depression in FBC 1987.

b Self-reported violent behaviors in RADAR, self- and mother-reported violent behaviors in ALSPAC, and official register of violent convictions in FBC 1987.

c Mean age of depression diagnosis followed from 1998 to 2012 (i.e., age 10–26 years).

d n (%).

e Mean age of the first violent convictions for individuals with and without depression, based on follow-up data from 2002 to 2014 (i.e., age 15–27 years).

In the RADAR and ALSPAC cohorts, the mean scores of depressive symptoms at age 13 years were significantly lower in boys than in girls (*t* [*df* = 610] = 3.6, *p* < .001 in RADAR and *t* [*df* = 3481] = 6.4, *p* < .001 in ALSPAC). Across these two cohorts, boys were more likely to commit violent behaviors than girls (35.2% boys vs. 23.0% girls, $\chi_{1}^{2}$ [N = 623] = 10.9, *p* < .001, phi coefficient [φ] = 0.1 in RADAR; 43.1% boys vs. 29.1 girls, $\chi_{1}^{2}$ [N = 4,030] = 84.2, *p* < .001, φ = 0.1 in ALSPAC).

In the FBC 1987 cohort, the mean age at the first outpatient diagnosis of depression was 19.7 years (SD = 3.5). The overall prevalence of depression was 3.6% and boys were less likely to be diagnosed with depression than girls (2.2% boys vs. 5.0% girls; $\chi_{1}^{2}$ [N = 57,526] = 310.0, *p* < .001, φ = −0.1). The overall rate of convictions for violent crime during follow-up was 3.7%, with boys more likely to be convicted of violent offense than girls (6.2% boys vs. 1.2% girls, $\chi_{1}^{2}$ [N = 57,526] = 917.3, *p* < .001, φ = 0.1). Moreover, 146 (7.1%) individuals with depression (97 male [14.5%] and 49 female [3.5%]) were convicted of one or more violent crimes, compared with 1,981 (3.6%) general population controls (1,687 male [5.8%] and 294 female [1.1%]).

### Longitudinal Effects of Depressive Symptoms and Diagnosis on Later Violence

Across cohorts, there were positive associations between measures of depression and subsequent violent behaviors, adjusted for the effects of family socioeconomic status and previous violence (Table 2). An overall adjusted odds ratio (aOR) of 1.7 (95% CI = 1.2–2.5) was reported in the RADAR cohort and 1.8 (1.4–2.3) in the ALSPAC cohort; that is, a 1-unit increase in the mean score of depressive symptoms was associated with 1.7- and 1.8-fold elevated risk of later violent behaviors, respectively. In the FBC 1987, the overall aOR of convictions for subsequent violent crime was 2.1 (1.7–2.7) among individuals with a depression diagnosis compared with general population controls. The full set of variables included in our regression models are presented on Table S1, available online.

**Table 2:** Longitudinal Association Between Depressive Symptoms or Diagnosis, and Subsequent Violent Behaviors in Adolescents and Young Adults

	Risk of Violence in Depression				
		cOR	95% CI	aOR	95% CI
RADAR	Overall	2.1	1.5–3.0	1.7	1.2–2.5
	Male	1.7	1.0–2.7	1.6	1.0–2.5
	Female	3.7	2.1–6.5	2.7	1.4–5.1
ALSPAC	Overall	2.1	1.7–2.6	1.8	1.4–2.3
	Male	2.2	1.5–3.2	1.8	1.2–2.6
	Female	2.5	1.9–3.4	2.2	1.6–2.9
FBC 1987	Overall	2.3	1.8–2.8	2.1	1.7–2.7
	Male	2.9	2.3–3.8	2.7	2.0–3.6
	Female	4.2	2.8–6.1	3.8	2.5–5.6

Note: Adjusted odds ratios (aOR) were adjusted for family socioeconomic status and previous violence. ALSPAC = Avon Longitudinal Study of Parents and Children; cOR = crude odds ratio; FBC = Finnish Birth Cohort; RADAR = Research on Adolescent Development And Relationships.

Across all three cohorts, higher aORs for violent outcomes were generally found in girls than in boys, although CIs were overlapping. In the RADAR cohort, an aOR of 2.7 (1.4–5.1) was reported in girls and 1.6 (1.0–2.5) in boys. In the ALSPAC cohort, the aOR was higher in girls (aOR [95% CI] = 2.2 [1.6–2.9]) than in boys (aOR [95% CI] = 1.8 [1.2–2.6]). In addition, in the FBC 1987, the aORs of violent convictions were higher for girls with depression (3.8 [2.5–5.6]) than for boys with depression (2.7 [2.0–3.6]), when compared to their same-gender population controls without depression. When we right-censored for death during follow-up, aORs did not materially alter (data not shown).

For FBC 1987, we performed additional analyses to test the risk of later violent convictions in individuals with an inpatient diagnosis of depression (n = 1,181), including those with and without an outpatient diagnosis of depression, compared to controls without depression (n = 55,477). The overall aOR of convictions for violent crime was 2.8 (2.2–3.7) among individuals with an inpatient depression diagnosis compared with general population controls without depression. In addition, the aORs of violent convictions were significantly higher for girls with depression (7.1 [5.0–10.2]) than for boys with depression (2.7 [1.9–4.0]), when compared to their same-gender population controls without depression.

Controlling for additional variables such as parental criminality, conduct disorder, and maltreatment made little difference to the findings. In RADAR, we controlled for maltreatment experiences including being a victim of sexual assault, a victim of physical assault, and threatened with violence, and we obtained similar results (i.e., aOR [95% CI] = 1.5 [1.0–2.3] in the total sample, 2.3 [1.1–4.6] in girls, and 1.4 [0.9–2.4] in boys). In ALSPAC, the odds of violence in depression remained similar after additional adjustment for parental criminality, conduct disorder, and childhood maltreatment (i.e., aOR [95% CI] = 1.8 [1.4–2.2] in the total sample, 2.2 [1.6–3.0] in girls, and 1.7 [1.2–2.6] in boys). In FBC 1987, results were similar after further adjustment of conduct disorder (i.e., aOR [95% CI] = 2.0 [1.6–2.6] in the total sample, 3.5 [2.3–5.2] in girls, and 2.5 [1.8–3.5] in boys). Furthermore, analyses with mean scores of depressive symptoms standardized in RADAR and ALSPAC revealed similar aORs. That is, 1 standard deviation of change in the mean score of depressive symptoms resulted in similar odds of violence in RADAR and ALSPAC (data not shown).

## Discussion

We found a consistent pattern of increased risk of later violent behaviors in depression in cohorts of adolescents and young adults in longitudinal analyses, with overall aORs ranging from 1.7 to 2.1 across all three cohorts. We also found that the odds of violent outcomes were typically higher in girls (aORs ranged from 2.2 to 7.1) than in boys (aORs ranged from 1.6 to 2.7). At the same time, the absolute risks in girls were considerably lower across the three cohorts.

Previous research has shown high rates of depression in adolescents in detention and correctional facilities.^10^ However, it has been difficult to generalize such findings to the general population, as the temporal relationship between depression and serious criminality is unclear in such cross-sectional investigations. Our longitudinal design allowed us to control for previous violence, enabling us to test whether adolescent depression is associated with changes in violence over time. This is a clear improvement, and we report similar findings despite geography, differences in measurements of exposures and outcomes, and increased violence risk by severity. This could potentially act as a foundation to test in trials of whether treating depression in younger persons can reduce crime outcomes. Moreover, our findings are in line with previous studies in adult samples showing links between depression and violence.^2^, ^4^ Although the associations between depression and violence reported here were slightly weaker than previous longitudinal studies in adults,^2^, ^4^ this may be a consequence of higher base rates of mild depression reported in younger persons.

Across all three cohorts, there was a trend that the odds of violent behaviors were higher for girls than for boys, although CIs overlapped. These findings are in keeping with research into risk of violence in bipolar disorders^26^ and personality disorders,^27^ and can be explained by the low absolute risks of violence by women. In other words, when the base rate is very low, any positive association leads to high relative risks, even when the absolute risk difference remains small. As part of any risk communication, clarifying both relative and absolute risks needs to be considered.

Our study has several strengths. First, we adopted a multiple-sample approach by using data from three different countries to examine the link between depression and violence, which adds to the generalizability of our findings. Second, we assessed depression and violence using different methods. In the RADAR and ALSPAC cohorts, the approach was potentially more sensitive, because we used continuous symptom measures and adopted questionnaires to assess violent behaviors. In the FBC 1987 sample, we investigated clinical diagnoses of depression primarily in outpatients but also in inpatients for secondary analyses, and extracted convictions for violent crime from a comprehensive national register. Using these multiple approaches, we demonstrated similar significant associations between depression and violence. In addition, we captured violent behaviors in adolescents who were below the age of criminal responsibility (i.e., 15 years) and therefore may not acquire a criminal record as a matter of law. Therefore, our study extends investigation to different phases of adolescence and early adulthood as well as testing levels of varying severity of violence. Third, all the cohorts were longitudinal, allowing us to test prospectively associations between depression and later violence and hence reduce the possibility of reverse causality bias.

The following limitations should be considered. First, our study used data from three high-income countries. The prevalence of adolescent depressive disorders among the three cohorts were comparable to the worldwide pooled prevalence.^16^ However, the rates of youth violence rates vary significantly among countries, with high-income countries reporting lower rates than low- and middle-income countries.^18^ Therefore, future studies of the depression–violence link in low- and middle-income countries^28^ are necessary. Second, different instruments were used to collect information on depression and violence across cohorts. Although the ORs in the three samples were highly comparable, they should be interpreted differently. Furthermore, direct comparison of results for different methods within cohorts was not possible. In addition, different measurements across studies will have contributed to between-sample heterogeneity. To further examine sources of heterogeneity, future research could consider pooling reported data or individual participant meta-analysis that synthesizes data using common definitions of variables.^29^ Third, no data on violence from other informants were available in the RADAR and FBC 1987 cohorts. Multi-informant data are recommended in future investigations to increase the validity of the measurements.^30^ Fourth, although our study provides evidence that depression predicts future violence, the possibility exists that depression and violence reinforce each other and, in doing so, increase their effects individually and in combination over time. For instance, it has been shown that aggression in adolescents may provoke peer victimization, which in turn leads to depression.^13^ Finally, although the rate of violent convictions in FBC 1987 was comparable to the national youth violence rate in Finland,^17^ ALSPAC and RADAR used community populations, and results reflect each local area. These two cohorts were mostly representative of the populations from which they were drawn, apart from possibly coming from a higher socioeconomic status based on house ownership in ALSPAC^31^ and employment type in RADAR.^32^ If these social class differences are validated, this would likely mean that our risk estimates are conservative, as lower social class is associated with violence outcomes.

The finding that depression in adolescents and young adults is associated with long-term violent outcomes has some potentially important implications for intervention and prevention. Although suicide risk assessment has been a standard clinical practice for individuals with depression,^33^ the risk of violence against others in depression has been largely neglected. Clinical practice guidelines in the United States^34^ very briefly discuss this link, which is not considered in UK National Institute for Health and Care Excellence (NICE) guidelines.^33^ If the findings are replicated and triangulated (with trials, for example), information on risk factors could be added. Furthermore, although the relative risk of violence in adolescent depression did not appear to be higher than in adult depression, the high absolute rates of violent behaviors and developmental salience of adolescent depressive symptoms suggest that consideration should be given to assessment and management of violence risk in this population. Compared with severe mental illnesses, such as schizophrenia and bipolar disorder, which are associated with violent behaviors,^35^, ^36^ adolescent and young adult depression is a more prevalent mental health condition. Furthermore, depression is chronic, recurrent, and appears to have long-term negative effects on violence. The relatively higher odds of violence in those individuals with an inpatient diagnosis of depression suggests that severity of depression may increase violence risk. Alternatively, it may indicate that inpatient episodes are triggered in part by violence risk. The former explanation, however, is consistent with the findings from two cohorts that used continuous measures.

Overall, this suggests, of course, that the timely treatment of depression is likely to decrease the subsequent risk of violent behaviors, and there needs to be more effort to this end. Thus, early identification and treatment of depression will have wide public health benefits. In addition, the findings that both depressive symptoms and clinical diagnosis were associated with future violence indicate that prevention of violence should be undertaken at both the population level in young persons with depressive symptoms who are not in contact with health care services, and in a targeted approach to a clinical population with specialist medical contact.

The potential importance of the risk management of individuals with depression in forensic settings, including juvenile detention and correctional facilities, is highlighted by these findings. As odds for violence are higher in girls, future research could focus more on addressing risk assessment and management for girls with depression.

Potential mediators and moderators of the reported links between depression and violence need clarification. The observed association may stem from shared mediators or moderators. These may include mediators such as impulsivity, hostility, and mood regulation^37^, ^38^ and moderators such as history of trauma and alcohol use.^39^, ^40^ Furthermore, the associations replicated across three different, relatively high-income countries suggest that common environmental factors such as community violence might moderate and genetic liability and stress sensitivity at an individual level might mediate the link between depression and violence.^12^ Future investigation of these potential mediators and moderators is needed. This information could assist in developing and testing interventions, and also could provide alternative treatment targets (e.g., impulsivity, mood instability, and recent victimization) in addition to treatment of the underlying depression.

This study has identified longitudinal associations between depression and later violent behaviors in three longitudinal cohorts. We found elevated risks of violence in adolescents and young adults with heightened depressive symptoms and in those with a clinical diagnosis of depression, and higher relative risks in girls than in boys. These findings highlight the need for improved treatment of depression in young people, clarification of mechanisms, and, if further validated, review of clinical guidelines.

## Figures and Tables

**Table S1:** Longitudinal Association Between Depressive Symptoms or Diagnosis, and Subsequent Violent Behaviors in Adolescents and Young Adults

	Risk of Violence in Depression		
	Overall OR (95% CI)^a^	Male OR (95% CI)	Female OR (95% CI)
RADAR			
Depression	1.7 (1.2, 2.5)	1.6 (1.0, 2.5)	2.7 (1.4, 5.1)
Prior violence	4.0 (2.7, 5.9)	4.0 (2.5, 6.5)	3.2 (1.7, 6.0)
SES	1.7 (0.9, 3.2)	1.5 (0.6, 3.9)	2.3 (0.9, 5.6)
ALSPAC			
Depression	1.8 (1.4, 2.3)	1.8 (1.2, 2.6)	2.2 (1.6, 2.9)
Prior violence	3.1 (2.6, 3.8)	2.3 (1.8, 2.9)	3.7 (2.6, 5.3)
SES	1.1 (1.0, 1.2)	1.0 (0.8, 1.2)	1.2 (1.0, 1.4)
FBC 1987			
Depression	2.1 (1.7, 2.7)	2.7 (2.0, 3.6)	3.8 (2.5, 5.6)
Prior violence	24.1 (20.9, 27.7)	16.7 (14.3, 19.5)	25.2 (16.0, 39.8)
SES	1.5 (1.4, 1.6)	1.6 (1.4, 1.7)	1.5 (1.2, 1.8)

Note: ALSPAC = Avon Longitudinal Study of Parents and Children; FBC = Finnish Birth Cohort; OR = odds ratio; RADAR = Research on Adolescent Development And Relationships; SES = socioeconomic status.

a ORs for violence were adjusted for the two other variables (e.g., the depression row refers to odds of violence in depression adjusted for prior violence and SES).
